# A sublethal dose of a neonicotinoid insecticide disrupts visual processing and collision avoidance behaviour in *Locusta migratoria*

**DOI:** 10.1038/s41598-017-01039-1

**Published:** 2017-04-20

**Authors:** Rachel H. Parkinson, Jacelyn M. Little, John R. Gray

**Affiliations:** Department of Biology, University of Saskatchewan, Saskatoon, SK Canada

## Abstract

Neonicotinoids are known to affect insect navigation and vision, however the mechanisms of these effects are not fully understood. A visual motion sensitive neuron in the locust, the Descending Contralateral Movement Detector (DCMD), integrates visual information and is involved in eliciting escape behaviours. The DCMD receives coded input from the compound eyes and monosynaptically excites motorneurons involved in flight and jumping. We show that imidacloprid (IMD) impairs neural responses to visual stimuli at sublethal concentrations, and these effects are sustained two and twenty-four hours after treatment. Most significantly, IMD disrupted bursting, a coding property important for motion detection. Specifically, IMD reduced the DCMD peak firing rate within bursts at ecologically relevant doses of 10 ng/g (ng IMD per g locust body weight). Effects on DCMD firing translate to deficits in collision avoidance behaviours: exposure to 10 ng/g IMD attenuates escape manoeuvers while 100 ng/g IMD prevents the ability to fly and walk. We show that, at ecologically-relevant doses, IMD causes significant and lasting impairment of an important pathway involved with visual sensory coding and escape behaviours. These results show, for the first time, that a neonicotinoid pesticide directly impairs an important, taxonomically conserved, motion-sensitive visual network.

## Introduction

Neonicotinoid insecticides (NIs) dominate a quarter of the global pesticide market^1^. Widespread NI crop treatment has increased overall pesticide use across North America^2^, despite growing evidence of toxic effects on wild and domestic bee populations^3, 4^, aquatic invertebrates^5–7^, and insectivorous birds^8^. NIs contaminate wetlands, via runoff and snowmelt from treated fields^9^, and wildflower and soil samples near treated fields at concentrations exceeding 10 ng/g^10^.

NIs are nicotine mimics, binding to invertebrate nicotinic acetylcholine receptor (nAChR) subunits^11^. Although commonly considered nAChR agonists, NIs also display antagonistic activity in some invertebrate species^7, 12^ and at certain doses^13^, suggesting that there are multiple nAChR binding sites^14^. Different NI compounds target different neurons and nAChR subtypes^15^ and NI metabolites may cause toxicity^16, 17^, accumulating at higher concentrations than the parent compounds^18^. For example, imidacloprid (IMD) has a 5 hour elimination half-life in honey bees and is completely metabolized within 24 h, while its metabolites persist for over 30 h^19^. In addition, IMD metabolites, including IMD-olefin, are more toxic than IMD^19^.

NIs display sublethal, neurotoxic effects in honeybees that range from altering food preferences^20^ to impairing navigation and foraging success^21–23^. Bee mushroom bodies have been a major focus of neonicotinoid research, given observed behavioural effects and speculation of a connection between NI use and colony collapse disorder. Chronic NI toxicity results in apoptosis and neuronal inactivation in the central nervous systems of bees and bats^24–26^ and markers of apoptosis have been shown to concentrate in the optic lobes of the honey bee within 24 hours of an acute dose^27^. Although generalized neuronal effects on the central nervous system have been demonstrated^24, 26^, the effects on an important visual networks has not been examined. The locust offers a highly tractable, ubiquitous system to directly address putative effects of NI toxicity on a well characterized motion sensitive network.

The locust nervous system has been studied extensively, initially in search of novel insecticide targets, and then increasingly as a system to investigate fundamental neural processes that control behaviour. Locusts display varied collision avoidance behaviours and possess tractable motion-sensitive visual neurons. Two of these neurons are the lobula giant movement detector (LGMD), which receives encoded visual information from retinotopic units (photoreceptors and corresponding optic lobe interneurons), and its postsynaptic partner, the descending contralateral movement detector (DCMD)^28^. The LGMD/DCMD pathway responds robustly to objects approaching on a direct collision course (looming)^29^, with peak firing rates occurring when the object surpasses an angular threshold on the retina^30^. These neurons also encode trajectory changes^31^ and maintain robust responses with the addition of complex backgrounds^32, 33^. The response profile of the LGMD/DCMD results from an interplay of excitatory and inhibitory inputs. Optic lobe interneurons transmit excitatory post synaptic potentials onto the dendritic field of the LGMD^34^. These synapses are nicotinic cholinergic, based on the presence of the enzymes for acetylcholine synthesis in the LGMD and retinotopic units^35^, and cholinesterase in the synapses^36^. Inhibitory synapses between neighboring retinotopic units (lateral inhibition) and between retinotopic units and the LGMD (feed forward inhibition) act to modulate and shape responses to object motion^36^.

The axon of each DCMD synapses monosynaptically with motorneurons in the thorax, including the fast extensor tibiae (FETi) and flight motorneurons in the meso- and metathoracic ganglia^34, 37^. DCMD action potentials evoke subthreshold EPSPs in these motorneurons^37–39^, and high-frequency DCMD spikes may summate sufficiently to cause spiking in motorneurons in which the membrane potential fluctuates due to activity in flight or in preparation for a jump^38, 39^. Bursting (brief intervals of high frequency spiking) of DCMD spikes occur in response to object approach and may play an important role in collision detection and generating escape behaviours^40^.

This tractable locust collision detection system provides a unique opportunity to examine the effects of a commonly used pesticide on visual sensory coding and visually-guided behaviours. Here, we show that an ecologically-relevant sublethal dose of IMD disrupts normal firing properties of the DCMD when the locust is presented with a looming stimulus, and that these disruptions are reflected by deficits in their ability to avoid collisions by flight steering or jumping. We show that behavioural effects are sustained 2 and 24 hours after an acute dose of IMD, and these correspond to significant decreases in peak DCMD firing rates within bursts. These findings support the hypothesis that collision avoidance behaviours depend critically on high DCMD firing rates within bursts. As DCMD-like neurons have been found in other invertebrate species, including the praying mantis^41^, crab^42^, and fruit fly^43^, these results have broader implications for invertebrates that rely on vision for navigation. For the first time, we show IMD directly impairs a visual motion detection pathway and collision avoidance behaviours at sublethal doses.

## Results

### LD50

**Figure 1:**
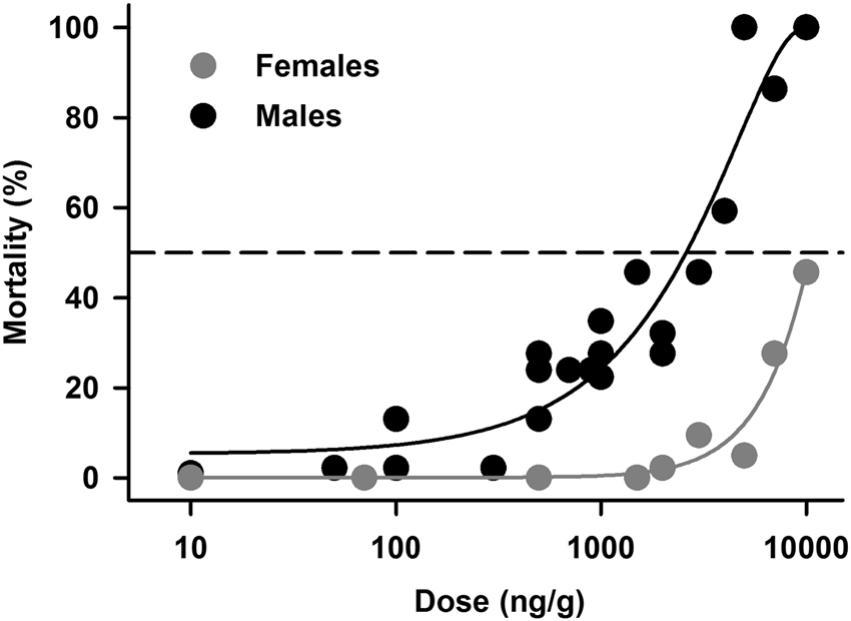
Percent mortality at 48 hours for male and female locusts after injection with imidacloprid (IMD) solutions ranging in concentrations from 10 to 10,000 ng/g (plotted on log scale), fitted with iterative non-linear regressions (solid lines). Points represent proportions from groups of 6 to 12 locusts, normalized to the mortality of vehicle control groups used on each testing day. Regression lines revealed an LD50 of approximately 2,500 ng/g for males. The LD50 for females was beyond the highest concentration used.

The 48 hour injected LD50 of IMD was determined with doses between 10 and 10,000 ng/g (ng IMD per gram of locust). At all doses ≥100 ng/g, locusts displayed increasing degrees of twitching, sporadic leg movements, and rapid abdominal movements, followed by periods of paralysis. After 48 hours, animals were scored as alive if they exhibited respiratory movements and moving mouthparts or legs. All male locusts treated with doses >1000 ng/g, and female locusts treated with doses >2000 ng/g were unable to walk in a coordinated manner at 48 hours, and were unresponsive. Percent mortality for males and females at 48 hours after treatment was normalized to the percent mortality in each sex of the vehicle controls. While females were not tested over the entire range of doses, they were more resistant than males to doses tested. Percent mortality was plotted against dose (Fig. 1), further illustrating the differences in sensitivity of males and females to IMD. We estimated the male 48 h LD50 at 2,500 ng/g, whereas for females it could be as high as 10,000 ng/g, although females were not tested over an adequate range of doses to confirm this. To prevent confounding results due to the large disparity in LD50 between males and females, we used male locusts for subsequent experiments.

### Behavioural Responses

**Figure 2:**
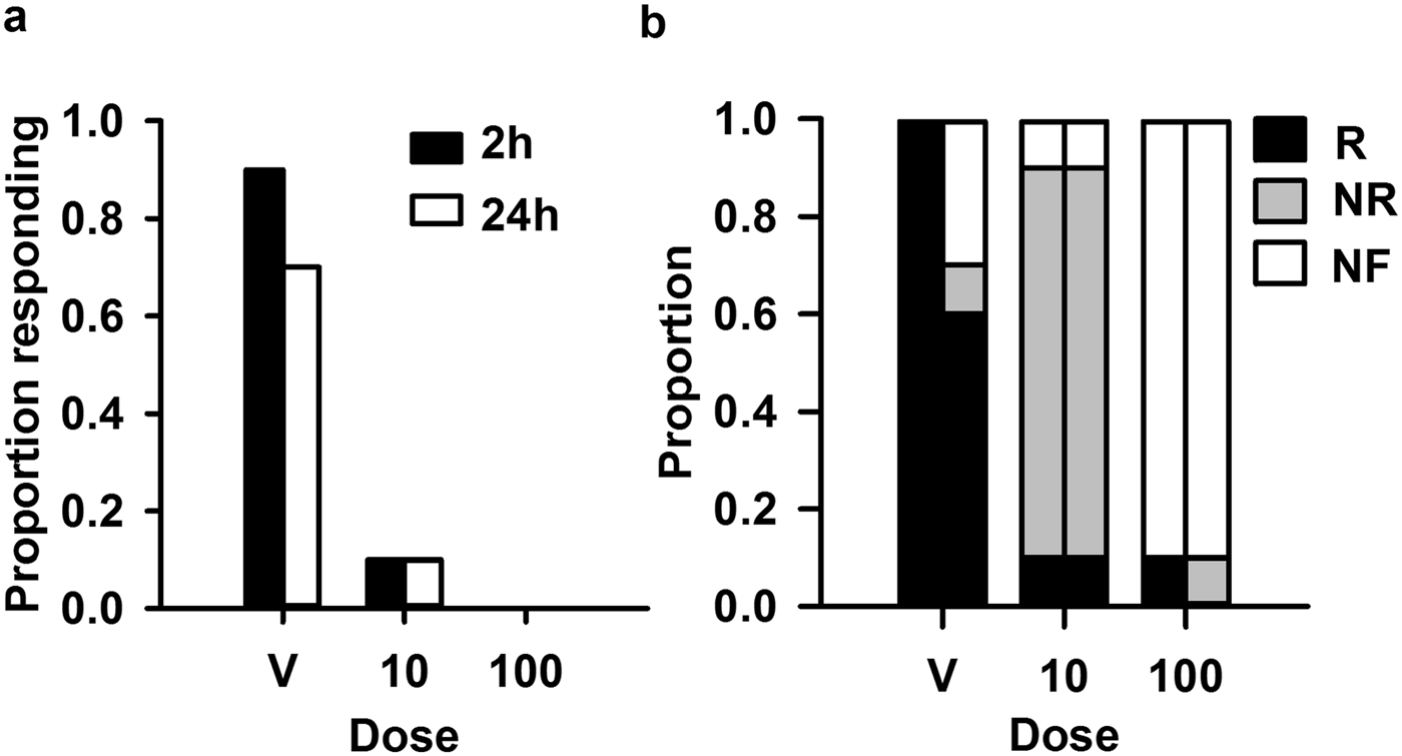
Effect of IMD on escape behaviours. (**a**) Proportion of animals responding to a looming stimulus by jumping or twitching hind legs at 2 and 24 hours after treatment with the vehicle, or 10 ng/g or 100 ng/g IMD. (**b**) Proportion of animals responding to looming stimulus while flying (R), flying but not responding to stimulus (NR), or not flying (NF) at 2 (left columns) and 24 (right columns) hours after treatment with vehicle or IMD.

We conducted behavioural assays on three groups of locusts treated with 10 or 100 ng/g IMD (n = 10 each), or the vehicle (n = 15). Behavioural assays took place in a wind tunnel with animals either standing (no wind) or flying (wind at 300 cm/s). We used a 14 cm black disk, approaching at 300 cm/s perpendicular to the orientation of the locust, which reliably evokes escape behaviours^44^. The effects of 10 and 100 ng/g IMD on escape behaviours persisted 2 and 24 hours after treatment (Fig. 2a). During jumping assays, none of the locusts treated with 100 ng/g reacted to the looming stimulus. For this assay, either a full jump or a twitch of the hind legs was scored as a reaction to the stimulus. Only one animal treated with 10 ng/g IMD responded to the looming stimulus at either time (twitch), while an average of 80% of vehicle controls reacted at both times (jump:twich ratio = 0.6:0.33 at 2 hours and 0.5:0.2 at 24 hours). IMD also affected locust flight behaviour (Fig. 2b). Most vehicle controls reacted to the looming stimulus with a collision avoidance behaviour (a glide, turn, or stop^44^) at 2 hours (100%) and 24 hours (60%) after treatment. Although 90% of the animals treated with 10 ng/g IMD flew at both 2 and 24 hours after treatment, only 10% responded to the stimulus and 90% of animals from the 100 ng/g treatment group did not fly. All escape behaviours (in flight and from standing) occurred between 2 and 0.2 s before the looming stimulus reached its largest size.

### DCMD Response (2 h)

To determine whether sublethal, ecologically realistic^45^ doses would affect DCMD responses to looming stimuli, we tested four IMD concentrations (0.1, 1.0, 10, and 100 ng/g), plus a vehicle (saline +0.2% acetone) on male locusts (n = 40) over 140 minutes each (Supplemental Table S1).

**Figure 3:**
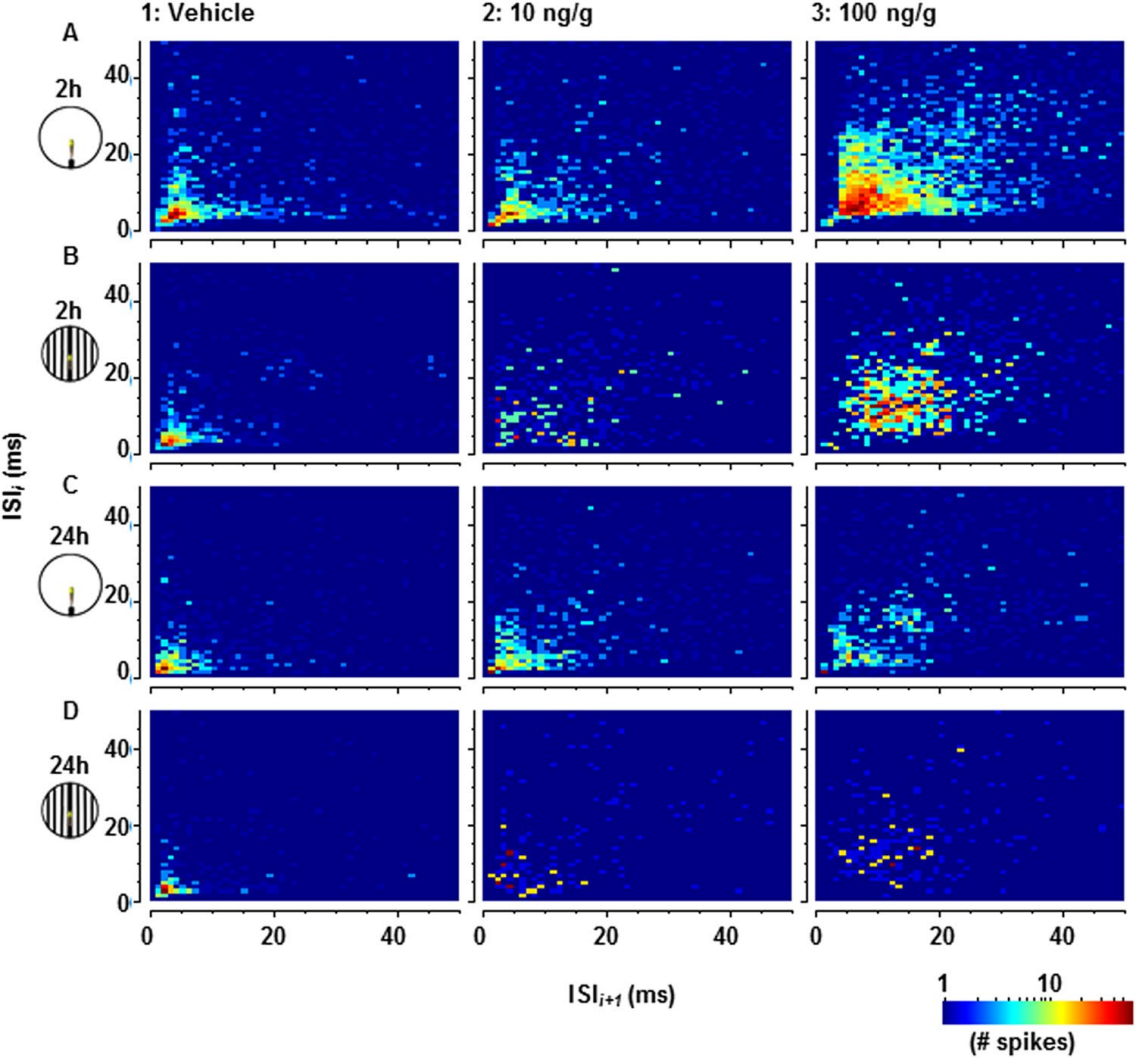
Joint inter-spike interval (ISI) distribution heatmaps comparing one ISI (y-axis, ms) with the following ISI (x-axis, ms). Heatmaps in column 1 were compiled from 5 stimulus presentations per animal either before treatment (1A,B, n = 25), or 24 hours after treatment with the vehicle (1C,D, n = 10). 2–3A,B were recorded from 80 to 110 minutes after treatment with 10 ng/g (n = 5) or 100 ng/g (n = 20) IMD. 2–3C,D were recorded 24 hours after treatment with 10 ng/g (2C,D, n = 10) and 100 ng/g (3C,D, n = 10).

**Figure 4:**
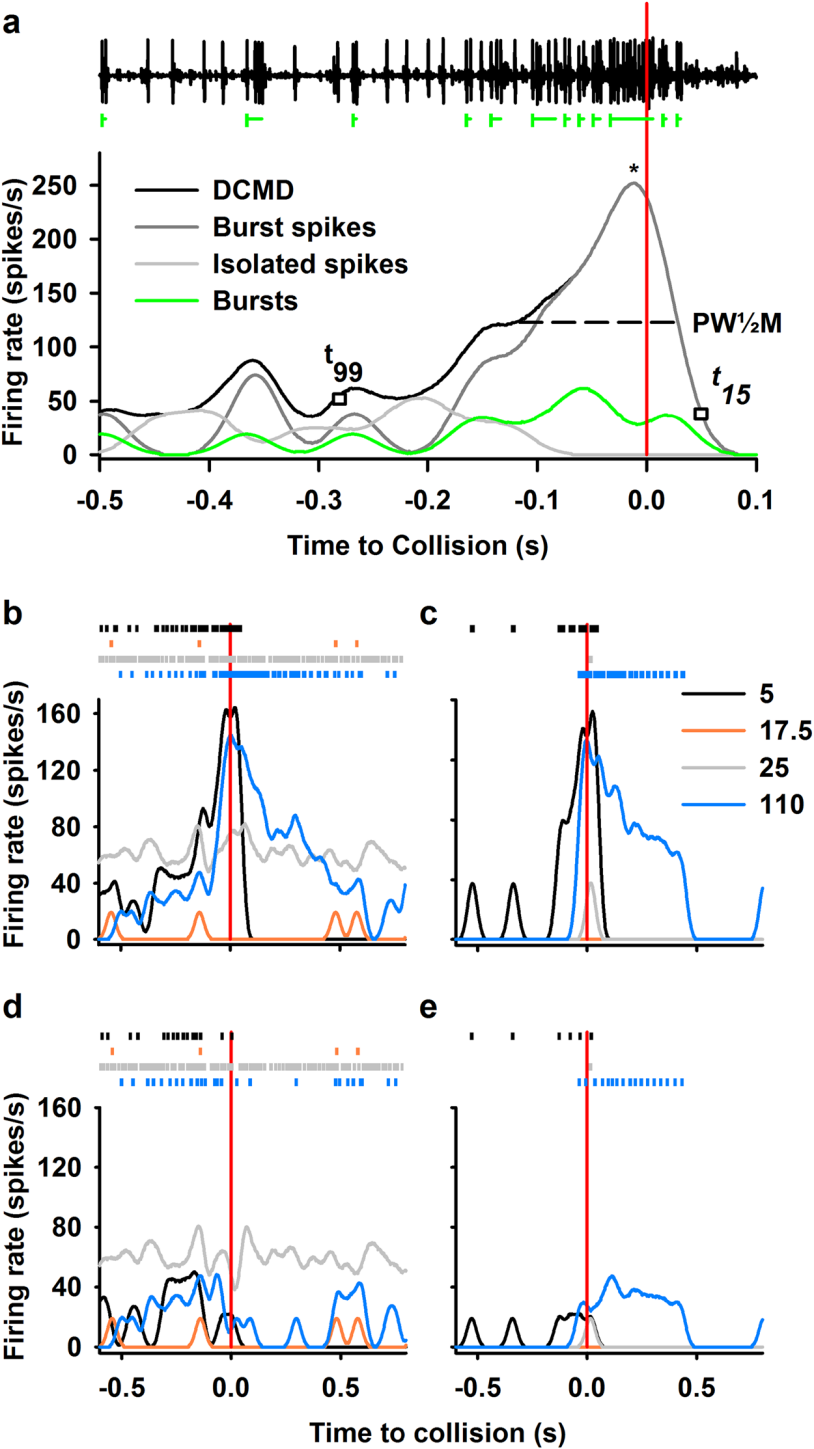
Rasters and peristimulus time histograms (PSTHs) constructed from DCMD responses to looming stimuli. (**a**) Raw continuous data from an extracellular recording of the left ventral nerve cord during presentation with the image of a 7 cm looming disk to the right eye (top). DCMD spikes were easily differentiated by their large amplitude. The projected time of collision (TOC) is marked with a vertical red line. Bursts, which comprise a minimum of two spikes occurring within 8 ms of each other, are highlighted with a vertical green line to signal the start, and horizontal lines to show the duration. PSTHs show the firing rates of the full DCMD response, spikes within bursts only, isolated spikes only, and bursts, smoothed with a 50 ms Gaussian filter. PSTHs response profile parameters included: peak firing rate (*f*_*p*_) and the time of the peak relative to TOC (pt), denoted by an asterisk; peak width at half maximum (PW½M), rise phase, from the last time the histogram crosses the 99% confidence interval with a positive slope (t99) to the peak, and decay phase from the peak until it had decreased to 15% (t15). (**b**–**e**) Show rasters (top) and PSTHs (bottom) at four time points after injection with 100 ng/g IMD, 5, 17.5, 25, and 110 minutes. These panels are separated by rate type: full DCMD (**b**), burst spikes only (**c**), isolated spikes only (**d**), and bursts (**e**).

The DCMD exhibits properties of bursting, with a minimum of 2 spikes and a maximum inter-spike interval (ISI) of 8 ms, and inter-burst intervals (IBIs) of 40–50 ms^40^. When presented with a looming visual stimulus, the frequency of DCMD spikes that are not contained within bursts (isolated spikes) is highest when the stimulus is farther away, while bursting frequency increases in the latter stage of the approach until collision^40^. It is likely that the timing of bursts is important for eliciting collision avoidance behaviours in flying locusts, in a process termed flight gating^39, 40^. To determine whether the presence of bursting was affected by IMD treatments, we constructed joint inter-spike interval (ISI) distribution heatmaps for DCMD responses (Fig. 3). In the presence of vehicle, ISIs clustered between 1–8 ms regardless of background or time, which is consistent with previously described DCMD bursting^40^. Against a simple background, ISI clusters were more broad (5–20 ms) 2 hours after treatment with 100 ng/g IMD whereas against a flow field at either dose and time or against a simple background 24 hours after treatment with 100 ng/g, clustering was less pronounced. Given this apparent disruption of bursting caused by treatment with IMD, full DCMD responses were subdivided by burst frequency, isolated spike frequency, and frequency of spikes contained within bursts (Fig. 4a). Measurements from the resulting peristimulus time histograms (PSTHs) were used to compare responses (Fig. 4a).

The effect of IMD was variable over the first hour, and stabilized through the second hour (Supplemental Fig. S2). We grouped the effect into four general phases (Fig. 4b–e): 1) pre-effect - within the first few minutes after injection (which resembled pre-treatment activity); 2) inhibition - a period of sporadic, low frequency firing, or complete neural silence; 3) high frequency, sporadic firing; and 4) post-effect - maintained as a stable response approximately 1 hour after treatment. During phases 1 and 4, most spikes were contained within bursts whereas for phases 2 and 3, most spikes were isolated. All animals tested with 10 or 100 ng/g IMD displayed phases 1 and 4 whereas phases 2 and 3 were less consistent.

**Figure 5:**
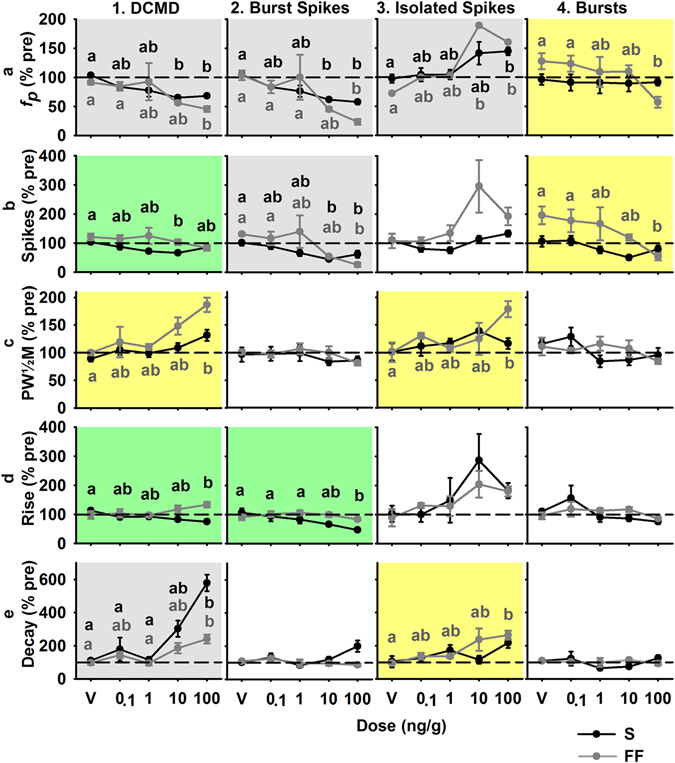
Response parameters (mean ± SEM) of the four DCMD rates (full DCMD, burst spikes, isolated spikes, and bursts) at 110 (S background) and 120 minutes (FF background) after treatment with an IMD dose (ng/g) or the vehicle (V). Data plotted as a percent of pre-treatment values within each animal. Grey backgrounds indicate a significant effect of one or more IMD dose for stimuli presented over both S and FF backgrounds, yellow indicates a significant effect within FF background only, and green for S background only. Significant results of post hoc analyses are indicated with letters colour coded to the respective background type.

Due to variability of IMD effects within the first hour of treatment, single time points were selected to compare between simple (S) and flow field (FF) backgrounds at 110 and 120 minutes after treatment, respectively. S represents a disc projected against a white background and FF represents a disc against a flow field consisting of vertically oriented, 2 cm wide bars moving outward from the azimuthal plan from the dome apex at 0.138 m/s. The latter elicits DCMD responses with lower peak firing rates, later peak time, shorter rise phase and a longer decay phase^32^. Doses of 0.1 and 1.0 ng/g did not significantly affect DCMD firing, while doses of 10 and 100 ng/g resulted in significant alterations of several features of DCMD firing (Fig. 5). Significant effects, for both backgrounds, included: a decreased peak frequency (*f*_*p*_) for DCMD and burst spikes (1, 2a); an increased *f*_*p*_ for isolated spikes (3a); a decreased number of spikes within bursts (4b); and an increased DCMD decay phase (1e). The total number of spikes within bursts also decreased. Parameters affected for S or FF stimuli may be attributable to the effects of these background types. Rise phases for the full DCMD response and for spikes within bursts were significantly shorter for S. However, the rising phase did not decrease with FF, though this time period is typically very short for FF due to the effects of lateral inhibition caused by the movement of the background across the visual field^46^.

### DCMD Response (24 h)

**Figure 6:**
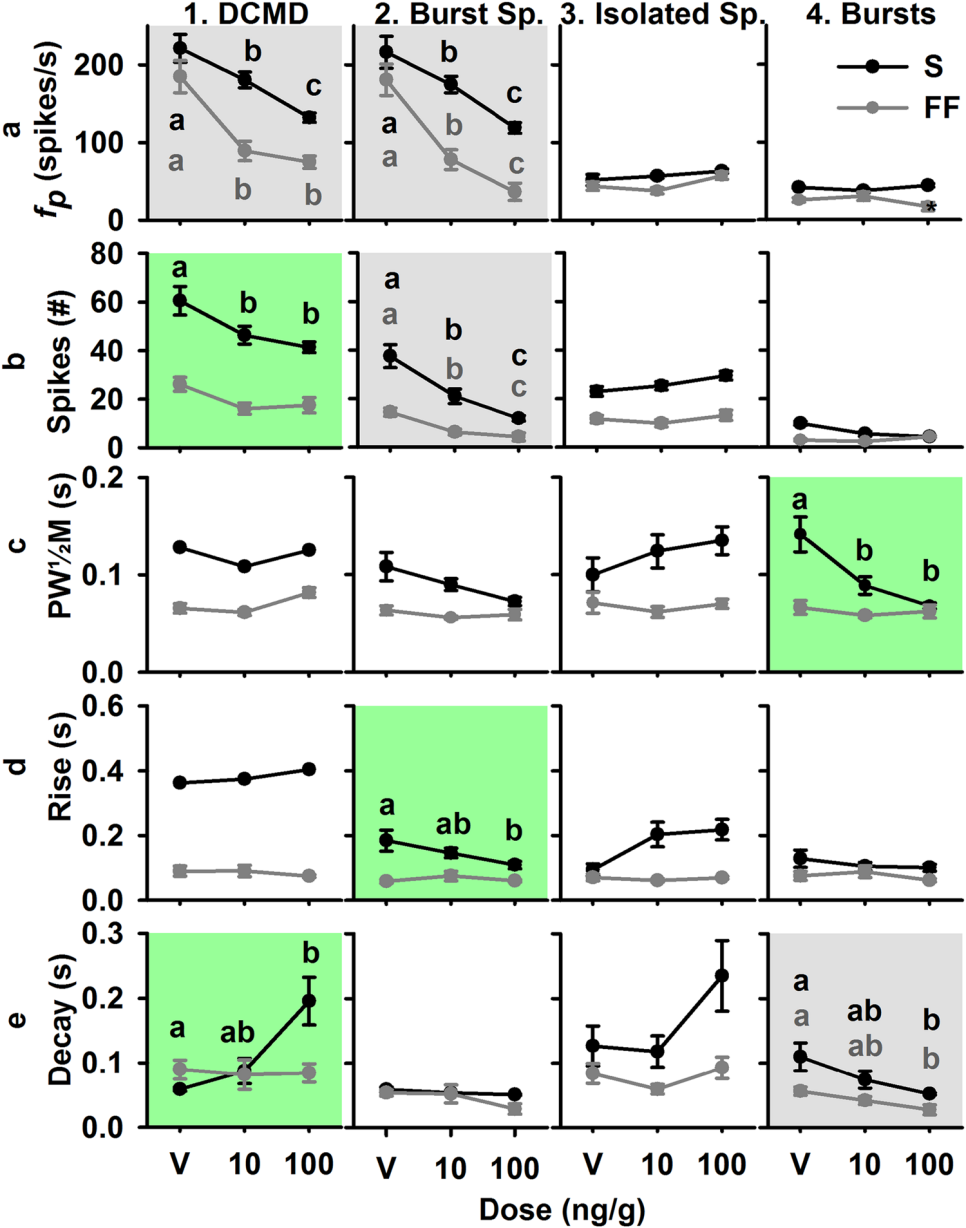
Response parameters (mean ± SEM) of the four DCMD rates (full DCMD, burst spikes, isolated spikes, and bursts) 24 hours after treatment with an IMD dose (ng/g) or the vehicle. Gray backgrounds indicate a significant effect of one or more IMD dose for stimuli presented against both S and FF backgrounds, and green indicates a significant effect with S background only. Significant results of post hoc analyses are indicated with letters colour coded to the respective background type.

**Figure 7:**
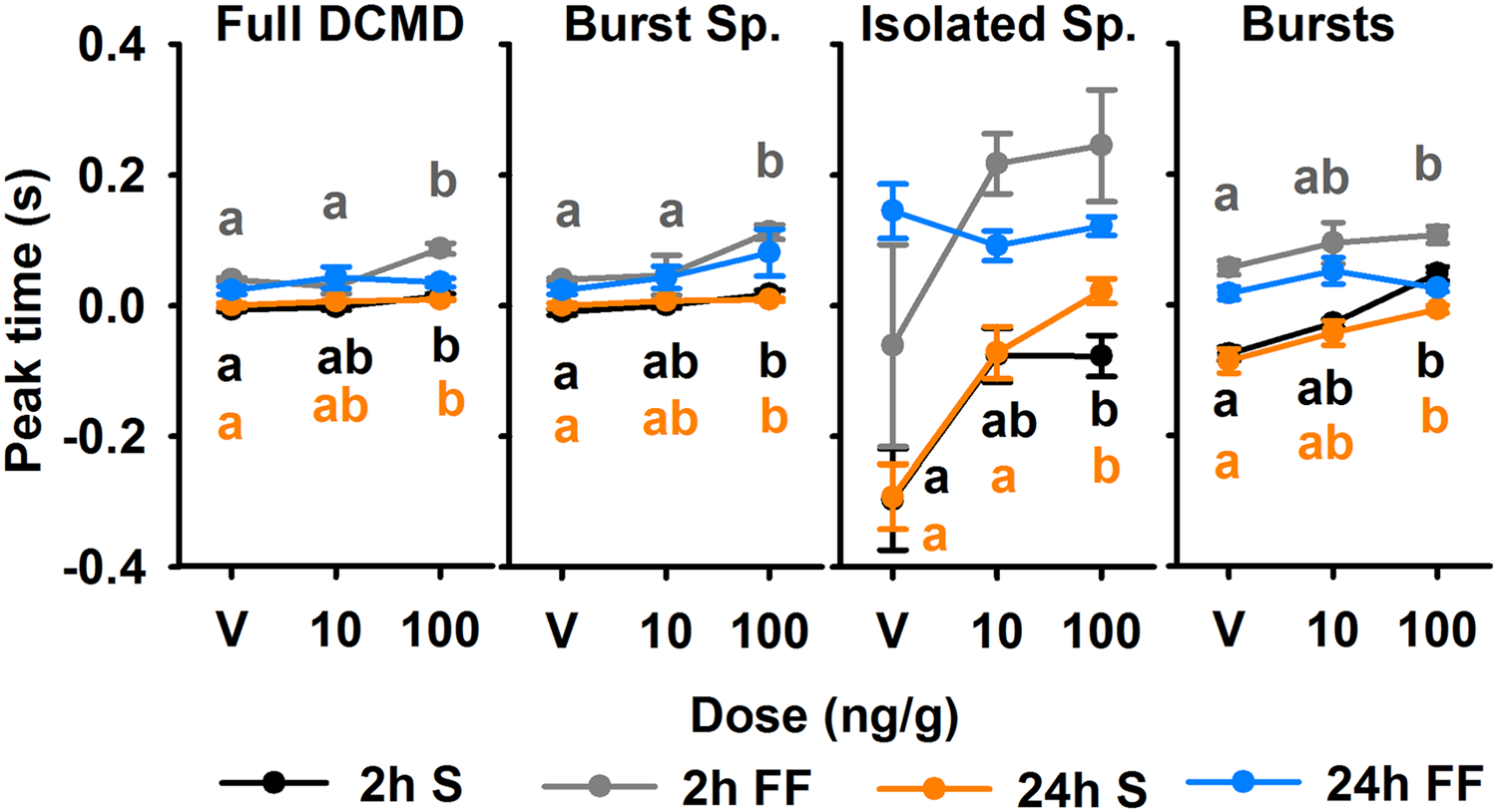
Time of maximum firing rate relative to collision (peak time; mean ± SEM) across doses and background types at 2 and 24 hours after treatment. Significant results of post hoc analyses are indicated with letters colour coded to the respective background type.

We were interested in whether IMD-induced alterations in DCMD firing persisted 24 hours after treatment, in accordance with behavioural deficits. Animals that participated in behavioural experiments were prepared for electrophysiology 24 h after initial treatment. We obtained successful recordings from 25 animals from behavioural assays. Peristimulus time histogram (PSTH) overlays of the 100 ng/g dose at 2 and 24 hours after treatment showed similarities in peak firing rates (Supplemental Fig. S3). We measured the same DCMD response parameters at 2 and 24 hours after treatment. Response variables significantly altered by IMD at 24 hours after treatment for both background types included the maximum firing rate (DCMD and burst spikes), the number of spikes (burst spikes), and the decay phase of bursts (Fig. 6). For stimuli presented over the S background, the number of spikes (DCMD), peak width at half height (bursts), rising phase (burst spikes), and decay phase (DCMD) were significantly altered by IMD treatment. The time of peak firing was significantly later relative to TOC at the 100 ng/g dose at both time points for S, as well as at 2 hours after treatment for FF (Fig. 7).

**Figure 8:**
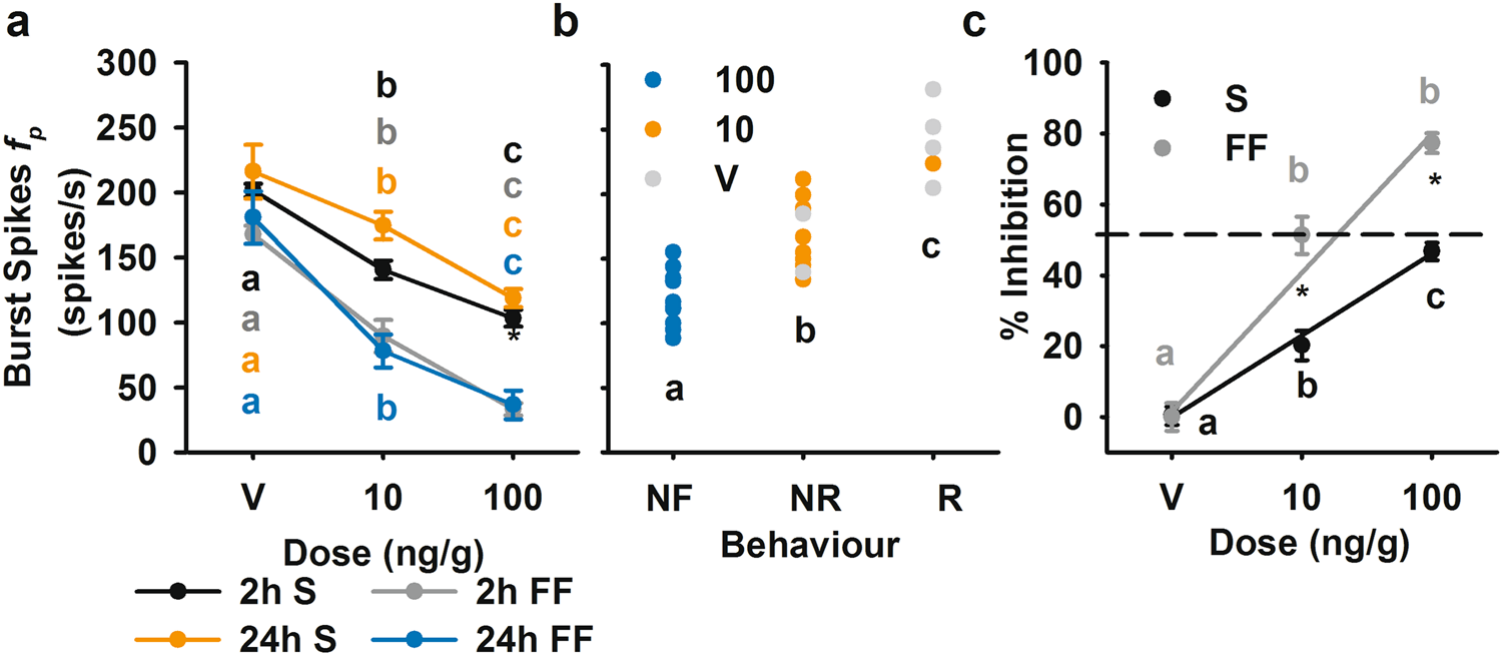
Effect of IMD on burst spike peak firing rate (*f*_*p*_) and correlation with observed behavioural deficits. (**a**) Burst spike peak *f*_*p*_ at 2 and 24 hours after treatment, for stimuli presented against simple (S) and flow field (FF) backgrounds. (**b**) Burst spike *f*_*p*_ correlated with flight behaviour at 24 hours after treatment with IMD or vehicle: NF, not flying; NR, flying but not responding; R, responding with a turn or glide, with a Pearson correlation coefficient of 0.872. Each data point represents measurements from a single animal, and letters denote significant differences between means. (**c**) Percent inhibition with effects at 2 and 24 hours combined for each background type, plotted as percent reduction from the mean of the vehicle treatment group. Colour coded letters denote significant differences between treatments within each background, and asterisks denote significant differences between S and FF within treatment.

To determine the overall inhibitory effects of IMD on DCMD firing, we chose to focus on the maximum firing rate of burst spikes (Fig. 8a). This is the only response variable that was significantly altered with 10 and 100 ng/g IMD, at both 2 and 24 hours after treatment, for stimuli presented over both S and FF backgrounds. There was no significant difference in the maximum firing rate of burst spikes 2 and 24 hours after treatment within dose and background type, while the burst spike firing rate significantly decreased with increasing IMD dose. Effects on peak burst spike firing rate are correlated with observed flight behaviours (Fig. 8b). Burst spike firing rate was inhibited to a greater degree with FF: 50% inhibition was achieved with a 10 ng/g dose with FF, while similar inhibition was achieved at the 100 ng/g dose for S (Fig. 8c).

## Discussion

We found that the 48 hour injected LD50 of IMD was 2,500 ng/g for male locusts. In the honeybee (*Apis mellifera*) the oral LD50 was estimated at 4.5 ng/bee (approximately 45 ng/g), and contact LD50 at 81 ng/bee (approximately 810 ng/g)^45^, although these values can differ greatly between colonies and studies^17^. While our reported LD50 is high it may be due to the delivery method, toxicological endpoint, and metabolism. Grasshoppers (*Melanoplus sanguinipes*) sustain a long period of “debilitation” before death, with high oral and contact LD50s of 53.38 and 96 ppm (µg/g), respectively, for IMD at 72 hours after treatment^47^. In honeybees, mortality is delayed from high doses of IMD, compared to moderate doses^48^, prompting use of a 96 h endpoint. Furthermore, suggestions to score insects as dead when moribund result from delayed insecticide toxicity^49^. Nevertheless, locusts are known to express a wide range of enzymes involved with xenobiotic detoxification^50^, and display resistance to many insecticides^51^. By injecting IMD into the hemolymph, it would bypass detoxification enzymes concentrated in the gut^51, 52^, and may not distribute naturally throughout the body. IMD metabolites are known to be toxic in other species, including honeybees^16, 17^, and can accumulate in high concentrations^18^. Metabolism of IMD could be slowed by injection into the hemolymph and limited by diffusion into other tissues. Additionally, locust hemolymph contains the lowest concentration of glutathion s-transferase enzymes, which are involved in pesticide metabolism^53^. The results of the present toxicity tests are not for direct comparison with values from oral or topical application, but provide a basis for scaling the doses we used in behavioural and electrophysiological assays. Interestingly, females displayed a four-fold higher resistance than males to IMD. These results were unexpected as individual females had masses two to three times greater than males, thus receiving a larger volume of IMD solution. Female locusts are more resistant than males to topically applied organophosphate and organochloride pesticides^54, 55^, although not at the high factor we report for injected IMD. Further studies are required to determine the cause of this difference between the sexes.

Initial effects of IMD on DCMD responses included periods of spontaneous activity, both high and low frequency firing, and/or periods of neuronal silence (hyperexcited and inhibited phases). These effects were pronounced at the 100 ng/g dose only, and commence within 10 minutes of treatment. Approximately 1 hour after treatment, the DCMD again responded to the looming stimulus in a stable manner (post-effect phase) that was maintained until the end of the recording period, two hours and 20 minutes after treatment. Acute behavioural effects of IMD toxicity include periods of trembling and sporadic movements of the abdomen and legs, followed by paralysis in the honeybee (*Apis mellifera*)^17^, flea (*Ctenocephalides felis*)^56^, beetle (*Tenebrio molitor*)^57^, and grasshopper (*Melanoplus sanguinipes*)^47^. Our results are consistent with other studies that found initial neuronal effects to be hyperexcitation followed by neural silence^57, 58^, which may be attributed to receptor desensitization^12, 59^.

The effects observed after the first hour of IMD toxicity could result from lingering upstream receptor desensitization. Debilitation of interneurons that converge onto the LGMD would limit the peak firing rate and number of spikes of the LGMD/DCMD by reducing the cumulative effect of EPSPs evoked by their inputs. The rising phase was shortened under the simple background only, likely because this period is already extremely short for flow field under normal conditions. The rising phase is associated with the threshold of the LGMD and DCMD^60^, and could be shortened by effects on excitatory inputs, and down regulation or impairment of nAChRs on the LGMD/DCMD. Decreased DCMD firing rates may also be attributed to reduced excitatory firing of the retinotopic units that synapse with the LGMD^60^, while increased isolated spikes *f*_*p*_ and lengthened decay phase may result from weakened feed forward and lateral inhibition^61^.

Differences in the responses of the DCMD after the first hour of toxicity may be attributable to toxicity of IMD metabolites^16, 17^. Of these, 5-Hydroxy-IMD and IMD-olefin are known to accumulate at higher concentrations than IMD in honeybees^18^, suggesting their excretion half-life is much longer than for IMD. The olefin metabolite is more toxic than pure IMD^17^ and accumulates in nAChR-rich tissues. This metabolite may also bind to nAChRs^19^.

At 24 hours after treatment, any effects of IMD on the rising and decay phases of the DCMD response to a looming stimulus have been eliminated. For presentations using the simple background, the number of spikes were reduced and the time of the maximum burst rate was later relative to TOC. Peak time of the DCMD relates to both the visual acuity of the locust and feed-forward inhibition from the photoreceptors in the optic lobe^36^. Here, we show that peak time was significantly later at the 100 ng/g dose at both 2 h with S and FF, and 24 h with S. Several parameters, including number of DCMD spikes and spikes within bursts, and peak time of bursts is significantly altered for S only at 24 hours, likely because of IMD effects being masked by flow field stimulation.

A single DCMD response variable, maximum firing rate within bursts, coincided with the effects of IMD on collision avoidance behaviours at 10 ng/g. The elimination half-life of IMD is 5 h in the honeybee^19^, so the reduced burst spike firing rate at 24 h suggests there was impairment of the DCMD and upstream network that was sustained after IMD has been metabolized. This may result from down regulation or damage to nAChRs caused by IMD metabolites, or whole neuron damage or apoptosis in cholinergic interneurons of the optic lobes. Low dose IMD toxicity has been shown to cause apoptosis in the central nervous systems of bats and honeybees^24, 27^. In bees, markers of apoptosis are visible within 1 day with doses 1/10th of the LD50, and these are more concentrated in the optic lobes than other areas of the CNS^27^. Our results suggest that low doses of IMD cause lasting damage to the visual network of locusts, which may inhibit detection of looming stimuli by affecting visual processing in the optic lobes.

The effect of IMD on the burst spike firing rate was more profound for FF than S. This further suggests that visual processing was reduced as the looming stimulus was somewhat obstructed by the vertical bars of the FF. With reduced visual processing, the expanding edges of the stimulus were more difficult to distinguish. Any damage to the optic lobes or photoreceptors would enhance the masking effect. For these reasons, free flying animals may experience an even greater visual deficit with lower IMD doses than those tested in tethered conditions. When comparing burst spike firing rates in the different treatment groups, we found that a reduction in rate corresponded with deficits in collision avoidance behaviour. It has previously been suggested that a threshold of burst spike firing rate is required to elicit a collision avoidance behaviour^38–40^, and we provide additional evidence to this effect. However, we did not perform behavioural assays and electrophysiology simultaneously. Nevertheless, burst spike firing rate was the only parameter significantly altered across doses and times.

The bursting algorithm used on the data is based on the results of McMillan and Gray^40^ who found frequent DCMD ISIs occur at 1–8 ms. Our data show that low, sublethal doses of IMD disrupts bursting, with ISIs either clustering at larger intervals or not clustering. ISIs define intra-burst firing rates (burst spike firing rate), and longer ISIs naturally result in lower peak rates. This effect was clear at the 100 ng/g dose with both backgrounds, while at the 10 ng/g dose it was more apparent with a flow field background, which mimics whole-field optic flow produced by self-motion. Given these results, bursting would likely be disrupted in free-flying animals at very low IMD doses.

The larger stimulus was used for behavioural experiments (14 cm versus 7 cm for electrophysiology) to elicit collision avoidance behaviours more reliably^62^. Regardless, stationary animals treated with 100 ng/g IMD do not respond at all to looming stimuli, and only 10% of animals tested with 10 ng/g responded. A jump is initiated in the locust when flexor muscles are released after a period co-contraction of flexor and extensor tibiae muscles^63^. Fast extensor tibiae (FETi) motorneurons receive monosynaptic input from the DCMD^64^. The DCMD does not directly initiate a jump, as EPSPs from the DCMD in FETi motorneurons summate, but do not result in an action potential^38^. Rather, the DCMD has a role in timing the movement in an animal that is already prepared to jump^38^. Lack of summation of EPSPs in the FETi motorneurons resulting from a low DCMD firing rate (Fig. 6) may explain the absence of jumping responses in locusts treated with IMD. These animals may also be unprepared to jump due to debilitation of the motor control of the flexor and extensor muscles, and input from the DCMD would be ineffective.

At 10 ng/g IMD, flight motor circuitry was minimally affected, as 90% of test subjects could fly. Nevertheless, only one animal from this group responded to the looming stimulus. The DCMD makes excitatory synapses with flight motorneurons in the meso- and metathoracic ganglia^37^. Bursts of spikes in the DCMD result in summating EPSPs in flight motorneurons that alone do not result in an action potential^37, 39^. DCMD frequencies >150 Hz produce EPSPs in flight motorneurons that result in an action potential during flight, as the electrical potential of the motor neuron membrane oscillates, resulting in flight-gating^39^. By this mechanism, the DCMD directly initiates collision avoidance behaviours in flight. Thus, the reduction in firing rate caused by 10 ng/g IMD may result in an inability of EPSPs from the DCMD to summate and produce a glide, turn or collision avoidance behaviour. With 100 ng/g, 90% animals were unable to fly suggesting that IMD affects the collision avoidance pathway at lower doses than those required to disrupt flight. Similarly, neonicotinoids are known to disrupt motor function in bees^65^, and result in dose-dependent induction of trembling and paralysis in many invertebrates^48, 56, 66^. The central pattern generator for locust flight is under muscarinic cholinergic control^67^, and the innervation of muscles is glutamatergic^68^, so disruptions of these pathways by IMD is unlikely. Further studies are required to understand the location of these effects in the central nervous system, or to determine if IMD or one of its metabolites may affect neurons that are not under nicotinic cholinergic control.

We have shown that locusts are highly resistant to IMD, and that this resistance differs between males and females. Further investigation is required to determine why females can sustain such elevated doses, and if this difference is maintained with oral or contact toxicity. Despite the ability to survive high doses of injected IMD, we show that low doses (0.4% and 4% of the LD50) significantly disrupt the response of a looming-sensitive neuron, and that this disruption has downstream effects on collision avoidance while flying and stationary. Our results strengthen the hypothesis that high frequency DCMD burst spike firing is required to elicit a collision avoidance behaviour^38, 39^, as this is the only firing parameter that is significantly affected under all testing conditions in animals that cannot perform collision avoidance behaviours. Deficits in burst spike firing can be related to damage to the upstream network, and thus, our findings suggest that low, acute doses of IMD results in damage to the optic lobe and motion detection neurons in the locust. More broadly, this research offers insight on sublethal effects of IMD on behaviours observed in non-target insects, as it suggests that IMD can cause significant and lasting impairment of visual circuits in the optic lobes. Mechanisms and extent of this damage will be an important focus of future studies.

## Materials and Methods

### Animals

Adult locusts (*Locusta migratoria*) were selected at least 2 weeks past the last imaginal moult, and reared on a diet of wheat grass and bran in a crowded colony in the Department of Biology, University of Saskatchewan, Canada, at 25–30 °C, with a 12 h light:12 h dark cycle. Males were used for behavioural assays (n = 35) and electrophysiology (n = 65), while both males and females were used for the LD50 tests (n = 226). All locusts were weighed prior to experimentation to adjust injected dose by weight. Median locust weight was 1.4 g (range = 1.1 to 1.9 g) for males, and 2.5 g (range = 1.6 to 3.6 g) for females.

### Solutions

Imidacloprid (IMD; Pestanal, Sigma-Aldrich, Oakville, Ontario, Canada) was dissolved in acetone (100% v/v) and diluted with locust saline (147 mmol NaCl, 10 mmol KCl, 4 mmol CaCl_2_, 3 mmol NaOH, 10 mmol Hepes, pH 7.2) to produce final concentrations ranging from 0.1 ug/ml to 10 mg/ml. Final solutions were adjusted to each contain 0.2% (v/v) acetone. A solution containing locust saline and 0.2% acetone was used as the vehicle control in all experiments.

### LD50

Prior to tests, animals were given ad libitum access to food. A total of 160 male and 66 female animals were used in LD50 assays over 22 replicates. 16 IMD doses between 10 and 10,000 ng/g were used. A vehicle control group was included on each trial day. Animals were treated by injecting 1 μl solution per gram of body mass through the lateral cervical cuticle. After 48 hours, the proportion of deceased animals was calculated, and this was normalized to the proportion of deceased animals in the vehicle group. Percent mortality was plotted against the logarithm of the dose to allow estimation of the 48 h LD50 with a fitted curve.

### Wind tunnel

A 0.9 × 0.9 × 3 m Plexiglass wind tunnel was utilized for behavioural assays. A rear projection screen, mounted on the right side of the wind tunnel, displayed the visual stimulus: the image of a 14 cm black disk, approaching the animal at 300 cm/s on a direct collision course. The stimulus was created with Vision Egg visual stimulus generation software^69^ on a Python programming platform. For jumping assays, locusts were oriented 15 cm from and parallel to the rear-projection screen so that the center of the stimulus was in line with the center of the eye and wind was not used. For flying assays, locusts were loosely tethered with fishing line 35 cm from the centre of the stimulus and wind speed was set to 3 m/s, which is comparable to a locust’s natural flight speed (3–6 m/s)^70^. To capture 3D movement of the locusts, two GoPro HERO4 Black (GoPro, Inc., San Mateo, California, United States) cameras were mounted 33° apart, 114 cm behind the animals, set to 1080p, 120 frames per second.

### Behaviour assays

35 locusts participated 2 and 24 hours after injection with 10 (n = 10), or 100 (n = 10) ng/g IMD, or the vehicle (n = 15). A 3D-printed tether (0.2 g) was attached to the dorsal pronotum with VetbondTM Tissue Adhesive 1469SB (3 M Animal Care Products, St. Paul, MN, USA) to capture body orientation. Animals were loosely tethered to the roof of the wind tunnel with fishing line. Stationary animals were presented with three stimulus replicates at 5 minute intervals, and scored as responders if they jumped or twitched hind legs in response to at least one stimulus presentation. Suspended animals were scored as responding (R) if they reacted to at least one stimulus presentation by gliding or turning, not responding (NR) if they were flying but not reacting to the stimulus, or not flying (NF) if they were unable to fly.

### Flight simulator

Electrophysiological experiments were conducted with animals mounted in a flight simulator with a rear-projection dome, as described in Guest and Gray^71^. The stimulus was the image of a 7 cm black disk traveling at 300 cm/s, created with Vision Egg visual stimulus generation software^69^ on a Python programming platform and represented as a 1,024 × 1,024 pixel portable network graphics (png) file. Vision Egg code contained correction factors to account for the curvature of the dome screen, and the refresh rate was held at 85 frames/s. A 1.2-ms transistor-transistor logic pulse included in each video frame and the vertical refresh synchronization pulse (vsync) from the video card (NVIDIA GeForce4 Ti4200 128 MB) were recorded along with continuous neuronal activity. Neural activity was amplified with a differential AC amplifier (A-M Systems, model no. 1700, gain × 10,000) and sampled at 25 kHz. Data was recorded using an RP2.1 enhanced real-time processor (Tucker-Davis Technologies, Alachua, FL) with Butterworth filter settings of 5 kHz (low pass) and 100 Hz (high pass).

### Electrophysiology

Legs were removed and a rigid tether was affixed to the ventral surface of the thorax with beeswax. A square of ventral cervical cuticle was dissected to expose the paired ventral nerve connectives anterior to the prothoracic ganglia. The locust was transferred to the flight simulator where a silver wire electrode was hooked around the left connective. Petroleum jelly was used to insulate and protect the preparation from desiccation. A silver wire was inserted into the abdomen and connected to ground. The locust was then oriented dorsal-side up facing the apex of the dome. In this orientation, the head of the locust was 12 cm from the dome, with 0° being directly in front of the locust, and 90° directly perpendicular to the centre of the eye. Stimuli were presented at intervals of at least 2.5 minutes to prevent neural habituation.

### DCMD (2 h)

Experiments were conducted using both simple (S) and flow field (FF) backgrounds. Four sublethal doses of IMD (0.1, n = 5; 1, n = 5; 10, n = 5; and 100, n = 20 ng/g) plus the vehicle control (0.2% acetone in saline, n = 5) were injected after pre-treatment recordings. Recordings continued over 120 minutes, first at 2.5 minute intervals, then at 10 minute intervals over a simple background (Supplemental Table S1). At 100 minutes the background was switched to flow field and animals were presented with five stimuli at 2.5 minute intervals.

### DCMD (24 h)

Animals that participated in behavioural assays were promptly prepared for DCMD recordings approximately 24 h after treatment with 10 or 100 ng/g IMD or the vehicle. Animals were presented with the stimulus over S and FF backgrounds.

### Data analysis

Post-hoc analysis of continuous neuronal activity was completed in Offline Sorter (Plexon, Dallas, TX), where DCMD spike times were identified by threshold analysis. DCMD spike times, along with corresponding vsync pulse times and transistor-transitor logic pulses for each stimulus presentation were exported to Neuroexplorer analysis software (NEX Technologies, Littleton, MA). Burst analysis was performed on DCMD spike time data, with bursts defined as spike trains of at least two spikes with inter spike intervals (ISIs) of 2 to 8 ms, using an algorithm designed by McMillan and Gray^40^.

The last transistor-transistor logic pulse and corresponding vsync pulse were used to determine the time the last frame was drawn on the screen, and this was extrapolated to determine the time of collision (TOC) of the black disk had it continued traveling to the locust’s eye. The TOC for each stimulus presentation was used to align spike times in peristimulus time histograms (PSTHs) with a 1 ms bin width and smoothed with a 50 ms Gaussian filter. PSTHs were created using the full DCMD rate, spikes within bursts only, isolated spikes only, and initial spike from each burst only (Fig. 3A). DCMD firing properties were characterized by the maximum firing rate (spikes/s); time of the maximum firing rate relative to TOC (peak time); peak width at half the maximum rate (PW½M); total number of spikes during the stimulus presentation; rise phase, calculated as the time the PSTH last crosses its 99% confidence interval until the time of the peak; and decay phase, calculated as the time from the peak until the firing rate had decayed to 15% of the maximum. Matlab (MATLAB, R2016a, The MathWorks Inc., Natick, MA) was used for calculation of rise and decay phases. Video recordings were analyzed in GoPro Studio (GoPro, Inc., San Mateo, California, United States).

### Statistical analysis

Statistical analyses were performed using R (The R Foundation for Statistical Computing) and SigmaStat 3.5, and figures designed with SigmaStat 12.5 (Systat Software Inc., Richmond, CA, USA). DCMD response variables across doses were compared with One Way ANOVAs and Holm-Sidak pairwise multiple comparison procedures for parametric data, and Kruskal-Wallis One Way ANOVAs on Ranks with Dunn’s Method pairwise multiple comparison procedures for non-parametric variables. Effects of background type across doses was completed with Mann-Whitney Sum of Ranks tests (non-parametric) or student’s t-tests.

## Electronic supplementary material

Supplementary Information
