# Supplementary material for: A sublethal dose of a neonicotinoid insecticide disrupts visual processing and collision avoidance behaviour in Locusta migratoria
Source: Sci Rep. 2017 Apr 20;7:936. doi: 10.1038/s41598-017-01039-1 (PMC5430526; doi:10.1038/s41598-017-01039-1)
Supplement: Supplementary file 1 — Supplementary Information [file 41598_2017_1039_MOESM1_ESM.pdf]

Supplementary Information

A sublethal dose of a neonicotinoid insecticide disrupts visual processing and collision avoidance behaviour in Locusta migratoria

Rachel H. Parkinson, Jacelyn M. Little, John R. Gray

Supplemental Table S1: Experimental procedure for 2 hour electrophysiology experiments performed in a flight simulator with looming stimuli presented over simple (S) and flow field (FF) backgrounds.

# Stimulus presentations	Interval (minutes)	Background type
2	2.5	S
2	2.5	FF
	IMD injection	
12	2.5	S
7	10	S
5	2.5	FF

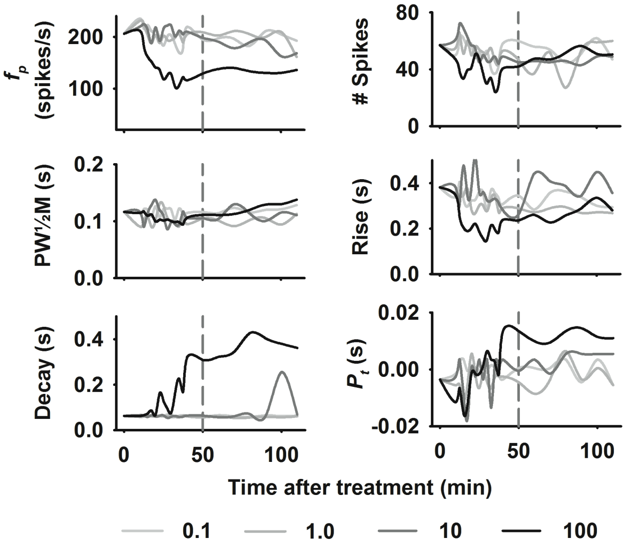

Supplemental Figure S2: Median temporal properties of DCMD response parameters over 100 minutes after treatment with 0.1 (n=5), 1 (n=5), 10 (n=5) and 100 (n=20) ng/g IMD during stimulus presentations against a simple background. Fifty minutes post injection is marked with a vertical dashed line, as the variability of responses at each dose is greatly reduced after this time.

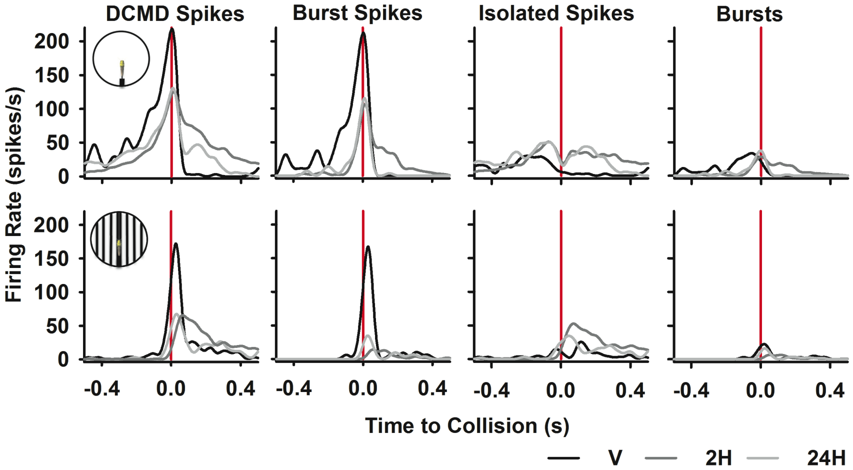

Supplemental Figure S3: Mean PSTH overlays of DCMD responses to a looming stimulus against simple (top row) or flow field (bottom row) backgrounds, at 2 (n = 20) and 24 (n = 10) hours after injection with 100 ng/g IMD. Responses of vehicle controls (n = 10) and untreated animals (n = 20) are displayed in black. Full DCMD response (left column) is subdivided in subsequent columns into burst spikes, isolated spikes, and bursts to show differences in these parameters at the two time points. Vertical red lines indicate TOC.

Supplemental Table S4: Results of statistical analyses for figures 5, 6, 7 and 8.

Resp. var.	DCMD Rate			Burst Spikes Rate			Isolated Spikes Rate			Burst Rate		
		Background			Background			Background			Background	
	Dose	S	FF	Dose	S	FF	Dose	S	FF	Dose	S	FF
**Fig. 5**												
**%Ymax**	U=210	19.3_4^{D}	18.9_4^{D}	U=204	21.6_4^{D}	25.0_4^{D}	U=376	16.5_4^{D}	4.9_4^{HS}	U=348	0.1_4	6.0_4^{H}
**%Spikes**	U=353	12.5_4^{D}	1.8_4	U=305	14.8_4^{D}	19.4_4^{D}	U=307	3.4_4^{HS}	$H=9.4_4$	U=432	$H=8.1_4$	17.3_4^{D}
**%PWHH**	U=227	$H=6.5_4^{D}$	16.5_4^{D}	U=403	$H=1.8_4^{D}$	13.7_4^{D}	U=287	0.7_4	13.8_4^{D}	U=410	$H=8.3_4$	9.7_4^{D}
**%Rp**	U=157	3.6_4^{HS}	2.0_4	U=166	8.5_4^{HS}	2.4_4	U=432	$H=9.1_4$	$F=2.4_4$	U=351	$H=7.4_4$	10.8_4^{D}
**%Dp**	U=171	28.7_4^{D}	16.5_4^{D}	U=226	$H=6.2_4$	$H=8.5_4$	U=327	H=7.5	15.8_4^{D}	U=424	H=6.3	$H=4.6_4$
**Fig. 6**												
**Ymax**	U=101	16.8_2^{HS}	18.8_2^{HS}	U=107	16.3_2^{HS}	25.4_2^{HS}	$t=10.7_{47}$	$H=5.9_2$	4.9_2^{HS}	U=89	$F=2.0_2$	$F=1.9_2$
**Spikes**	$t=9.1_{47}$	6.8_2^{HS}	1.6_2	U=84	16.0_2^{D}	6.0_2^{HS}	$t=9.3_{47}$	1.7_2	1.9_2	U=132	10.7_2^{D}	9.0_2^{HS}
**PWHH**	U=13	1.7_2	8.0_2^{HS}	U=51	7.4_2^{D}	$H=2.9_2$	U=65	$H=2.5_2$	$H=1.6_2$	U=69	13.2_2^{HS}	$F=0.2_2$
**Rp**	U=0	$H=3.0_2$	$H=0.4_2$	U=29	3.9_2^{HS}	$H=1.5_2$	U=44	3.4_2	H=5.2	U=109	0.8_2	$H=1.6_2$
**Dp**	U=227	8.1_2^{D}	H=2.3	U=136	3.4_2	H=5.7	U=151	2.5_2	$H=3.9_2$	U=96	11.1_2^{D}	3.8_2^{HS}
**Fig.7**												
**Pt 2h**	U=137	25.5_2^{D}	27.8_2^{D}	U=203	28.5_2^{D}	27.4_2^{D}	U=490	27.4_2^{D}	$H=12.7_2$	U=547	40.8_2^{D}	13.7_2^{D}
**Pt 24h**	U=51	25.5_2^{D}	32.8_2^{D}	U=30	28.5_2^{D}	31.8_2^{D}	U=134	27.4_2^{D}	12.7_2^{D}	U=12	30.8_2^{D}	13.5_2^{D}
**Fig. 8**												
**Ymax 2h**				$t=5.7_{47}$	69.9_2^{HS}	42.4_2^{D}						
**Ymax 24h**				$t=5.1_{28}$	14.0_2^{HS}	23.7_2^{HS}						
**Behav.**					16.3_2^{HS}							
**%Inhib.**				$t=7.1_{81}$	77.0_2^{HS}	60.0_2^{D}						

Columns are divided by rate type, and subdivided by dose or background. Rows under "Dose" compare between background types, and below each background (S and FF) compares between doses within each background. Values represent the H statistic from one-way ANOVA on Ranks test. Numerical subscripts indicate degrees of freedom. Post hoc tests denoted as Holm Sidak (HS) or Dunn's (D). Results from Mann-Whitney tests are indicated by the U statistic, and t-tests by the t statistic. Shaded cells indicate a significant effect ($P < 0.05$). Specific differences from post hoc tests indicated on Figures 5-8. Response variables (resp var): Ymax, peak firing rate; Spikes, number of spikes; PWHH, peak width at half height; Rp, rise phase; Dp, decay phase; Pt, peak time. %, percent of mean of control.
